# Recombinant truncated latency-associated peptide alleviates liver fibrosis in vitro and in vivo via inhibition of TGF-β/Smad pathway

**DOI:** 10.1186/s10020-022-00508-2

**Published:** 2022-07-16

**Authors:** Xudong Song, Jiayi Shi, Jieting Liu, Yong Liu, Yang Yu, Yufei Qiu, Zhiqin Cao, Yu Pan, Xiaohuan Yuan, Yanhui Chu, Dan Wu

**Affiliations:** 1 Heilongjiang Province Key Laboratory of Anti-Fibrosis Biotherapy, Mudanjiang Medical University, No. 3, Tongxiang Street, Aimin District, Mudanjiang, 157011 Heilongjiang China; 2 College of Life Sciences, Mudanjiang Medical University, Mudanjiang, 157011 Heilongjiang China; 3 Mudanjiang Medical University, Mudanjiang, 157011 Heilongjiang China

**Keywords:** Liver fibrosis, Transforming growth factor β1, Latency-associated peptide, TGF-β/Smad pathway

## Abstract

**Background:**

Liver fibrosis is a progressive liver injury response. Transforming growth factor β1 (TGF-β1) is oversecreted during liver fibrosis and promotes the development of liver fibrosis. Therapeutic approaches targeting TGF-β1 and its downstream pathways are essential to inhibit liver fibrosis. The N-terminal latency-associated peptide (LAP) blocks the binding of TGF-β1 to its receptor. Removal of LAP is critical for the activation of TGF-β1. Therefore, inhibition of TGF-β1 and its downstream pathways by LAP may be a potential approach to affect liver fibrosis.

**Methods:**

Truncated LAP (tLAP) plasmids were constructed. Recombinant proteins were purified by Ni affinity chromatography. The effects of LAP and tLAP on liver fibrosis were investigated in TGF-β1-induced HSC-T6 cells, AML12 cells and CCl_4_-induced liver fibrosis mice by real time cellular analysis (RTCA), western blot, real-time quantitative PCR (RT-qPCR), immunofluorescence and pathological staining.

**Results:**

LAP and tLAP could inhibit TGF-β1-induced AML12 cells inflammation, apoptosis and EMT, and could inhibit TGF-β1-induced HSC-T6 cells proliferation and fibrosis. LAP and tLAP could attenuate the pathological changes of liver fibrosis and inhibit the expression of fibrosis-related proteins and mRNAs in CCl_4_-induced liver fibrosis mice.

**Conclusion:**

LAP and tLAP could alleviate liver fibrosis in vitro and in vivo via inhibition of TGF-β/Smad pathway. TLAP has higher expression level and more effective anti-fibrosis activity compared to LAP. This study may provide new ideas for the treatment of liver fibrosis.

## Background

Liver fibrosis is a pathological change of chronic liver damage caused by various factors, such as alcohol consumption, viruses, and drug abuse, which can cause chronic liver inflammation and lead to an abnormal trauma healing response (Bolognesi et al. 2007; Parola and Pinzani 2019; Su et al. 2014). The primary manifestation is the abnormal deposition of extracellular matrix (ECM) components, which ultimately leads to the formation of fibrous scarring (Chen et al. 2020; Theocharis et al. 2019). Liver fibrosis is a reversible pathological response, and timely reversal of liver fibrosis can prevent chronic liver disease from progressing to cirrhosis and liver cancer (Hernandez-Gea and Friedman 2011). The pathophysiological process of liver fibrosis is a complex process regulated by multiple signaling pathways, of which the TGF-β1 signaling pathway plays a key role in the development of liver fibrosis (Lichtman et al. 2016; Xu et al. 2016). TGF-β1 is lowly expressed in normal liver cells. However, when the liver is injured, TGF-β1 is released to promote the activation of hepatic stellate cells (HSCs), which leads to the abnormal deposition of extracellular matrix, thus promoting the development of liver fibrosis (Ye et al. 2021; Zhang et al. 2016).

TGF-β1 is a protein synthesized from an inactive precursor and requires activation to function (Khalil 1999). The TGF-β1 proprotein consists of three parts: a signal peptide, an N-terminal precursor called a latency-associated peptide (LAP), and a C-terminal TGF-β1. The signal peptide is cleaved before secretion and the proprotein becomes inactive pro-TGF-β1 (Robertson et al. 2015). After cleavage by the Furin, the TGF-β homodimer binds with the LAP homodimer to form a small latent complex (SLC). SLC cross-links with latent TGF-β-binding protein (LTBP) to form the large latent complex (LLC). After the Arg-Gly-Asp (RGD) motif of LAP interacts with integrin αvβ6 or αvβ8, TGF-β1 is released from latent complex and activates its receptor. Upon binding to TGF-β1, TGF-βRII recruits and phosphorylates the intracellular domain of TGF-βRI, which subsequently activate downstream Smad signaling pathway (Shi et al. 2011).

LAP contains a straitjacket domain and an arm domain. The straitjacket is formed by the α1 helix and the latent lasso. The arm domain contains two four-stranded β-sheets consisting of eight β-strands (Shi et al. 2011). The α3 helix that connects the top and bottom strands is important for binding TGF-β1 and forming latent complex (Stachowski et al. 2020). β-strand folds β8 and β9 connect two arm domains in a bowtie tail. The RGD motif in the arm domain is essential for integrin binding and TGF-β1 activation (Dong et al. 2017). The α2 helix and most of the β strands are conserved in all the TGF-β family members. The Binding of TβRI to TGF-β1 is blocked by α1, α5 helix, fastener, β-strands β1, β3 and β10. The binding of TβRII to TGF-β1is blocked by the latent lasso (Shi et al. 2011). The straitjacket and the arm domain together complete the wrapping of TGF-β1. Removal of LAP is critical for TGF-β1 activation (Saharinen et al. 1999). Inhibition of TGF-β1 activation and receptor binding by LAP may be a potential approach to affect liver fibrosis.

In our previous study, the full-length PET28a-LAP recombinant plasmid was constructed and the LAP protein was purified by Ni affinity chromatography (Song et al. 2022). The soluble expression of LAP is low, which is not conducive to mass production and application. Therefore, based on the structural composition and function of LAP, seven truncated LAP plasmids were constructed to screen truncated LAP (tLAP) with higher expression levels, lower molecular weight, and comparable activity to LAP. The effects of recombinant LAP and tLAP on liver fibrosis were investigated in vitro and vivo experiments.

## Materials and methods

### Plasmid construction and protein expression

LAP is composed of 249 amino acids (Human TGF-β1 proprotein, P01137, 30-278). The truncated LAP fragments are No. 1 (1-132), No. 2 (1-65), No. 3 (1-100), No. 4 (45-132), No. 5 (77-249), No. 6 (77-193) and No.7 (194-249). Primers were designed and added with EcoRI and XhoI restriction sites. The target fragments were amplified by PCR and digested. The digested products were inserted into the PET-28a vector to construct recombinant plasmids with a His-tag at the N-terminus. The recombinant plasmids were transformed into *E. coli* C43(DE3) competent cells. Positive clones were cultured overnight at 37 °C in 3 mL LB medium. The bacterial liquid was inoculated into 20 mL of LB medium and cultured to an OD600 of 0.8. Then the bacterial liquid was added with 0.2 mM IPTG (Biofroxx, Germany) and incubated at 16 °C for 16 h. The collected cells were resuspended with 500 μL Buffer A (25 mmol Tris 7.5, 300 mmol NaCl, 2 mM PMSF) and sonicated on ice. The supernatant was collected by centrifugation and combined with 30 μL of Ni–NTA agarose at 4 °C for 1 h. 500 μL Buffer B (25 mmol Tris 7.5, 300 mmol NaCl, 15 mmol Imidazole) was added to wash Ni–NTA agarose for 3 times. Protein expression was detected by 12% SDS-PAGE.

### Protein purification and identification

The bacterial liquid cultured overnight was inoculated into 1 L LB medium at 1:200 and cultured at 37 °C for 4 h. Then the bacterial liquid was cooled to 16 °C and induced by 0.2 mM IPTG for 16 h. After sonication and centrifugation at 4 ℃, the supernatant was loaded onto a Ni–NTA column. Impurity proteins were removed by washing buffer containing a gradient of imidazole. The target proteins were eluted with Buffer C (25 mmol Tris 7.5, 300 mmol NaCl, 300 mmol Imidazole). The imidazole was removed by multiple dialysis and the buffer was replaced with PBS. The proteins were detected by 12% SDS-PAGE and pulldown assay.

### Cell culture

The hepatic stellate cell line, HSC-T6 (iCell Bioscience Inc, Shanghai, China) were cultured in DMEM medium (Cat.C11995500BT, Gibco, USA) supplemented with 1% penicillin/streptoycin (Cat. 15410, Gibco, BRL, USA) and 10% fetal bovine serum (FBS, Cat. P30-3302, PAN-Biotech, Germany). HSC-T6 cells were cultured at 37 ℃ in an incubator containing 5% CO_2_.

The hepatocyte line, AML12 cells (iCell Bioscience Inc, Shanghai, China) were culured in DME/F-12 medium (Cat.SH30023.01, HyClone, USA) supplemented with 1% penicillin/streptoycin and 10% fetal bovine serum. AML12 cells were cultured at 37 ℃ in an incubator containing 5% CO_2_.

### MTT and RTCA assay

The effects of recombinant LAP and tLAP on the proliferation of HSC-T6 cells induced by TGF-β1 were detected by MTT assay. Cells were plated in 96-well plates. HSC-T6 cells were treated with TGF-β1 (10 ng/mL, Pepro Tech, 100-21, USA), followed by the addition of 15, 30, and 60 μg/mL of LAP and tLAP, respectively. Absorbance was measured at OD490 after 24 h. Subsequently, the effects of recombinant proteins on the cell index of HSC-T6 cells were detected by RTCA. HSC-T6 cells were placed in E-Plate and TGF-β1 (10 ng/mL) was added. Recombinant LAP and tLAP were added, and the cell index was detected every 1 min for 72 h.

### Immunofluorescence assay

About 50,000 cells were added to each well of the 6-well plate with cover glass by cell slide method, and TGF-β1 (10 ng/mL) was added to each well after the cells adhered. 60 μg/mL of LAP and tLAP were added for intervention. The cells were fixed with 4% paraformaldehyde after 48 h. The cells were treated with 0.1% TritionX-100 for 20 min and then sealed with 5% BSA at room temperature for 1 h. Cells were incubated overnight at 4 ℃ with the following primary antibodies from Affinity Biosciences (Cincinnati, OH, USA): α-SMA (Cat.AF1032), Collagen I (Cat. AF7001), FN (Cat.AF5335) at a dilution of 1:200. The next day, fluorescent secondary antibodies (Cat.A0568, Beyotime, China) were incubated at room temperature and protected from light. Nuclei were stained with DAPI. Cells were observed by confocal laser microscopy.

### Animal experiments

Eight-week-old male C57BL/6 mice were purchased from Liaoning Changsheng Biotechnology Co., Ltd. (SCXK-2020-0001, China). The animal study protocol was approved by the Institutional Animal Care and Use Committee of Mudanjiang Medical University (IACUC-20210109-8). The mice were maintained at comfortable temperature on a 12 h:12 h light–dark cycle and 50–60% relative humidity. For the experiments, the mice were randomly divided into 4 groups. Control mice were injected with 1 mL/kg of olive oil twice a week for 8 weeks. The CCl_4_ group was given 1 mL/kg of CCl_4_ (prepared with olive oil, Cat. 56-23-5, Sigma-Aldrich, USA) twice a week for 8 weeks. The LAP and tLAP treatment group were administered CCl_4_ in the same way as the CCl_4_ group. At the same time, starting from week 7, mice were injected with LAP and tLAP proteins at a dose of 60 μg/mouse, twice a week for 2 weeks.

### Histopathological analysis

The liver tissue was immediately fixed in a 10% formaldehyde solution and gradient ethanol for dehydration treatment, then embedded in paraffin to make 5 μm thick tissue sections. Before pathological staining, the slices were placed in an oven at 60 ℃ for 2 h, then dewaxed with xylene and rehydrated with gradient ethanol. Tissue sections were then stained with H&E, Sirius Red (Cat.MM1004, MaoKang biotechnology Co., Ltd, Shanghai, China), Masson (Solarbio, Peking, China), and were performed to observe the structural changes and collagen fiber deposition of the liver tissues in each group by an optical microscope.

For immunohistochemical staining, the tissue sections were dewaxed and rehydrated with gradient ethanol. After antigen repair with hydrogen peroxide and sodium citrate, the tissue sections were sealed with 5% BSA at room temperature for 1 h. Tissue sections were incubated overnight at 4 ℃ with the following primary antibodies from Affinity Biosciences (Cincinnati, OH, USA): α-SMA (Cat. AF1032), Collagen I (Cat. AF7001) at a dilution of 1:200. After washing 3 times by PBS, the anti-rabbit IgG (Cat. ZB-2301, Zhongshan-golden bridge, China) secondary antibodies was incubated at 37 ℃ for 1 h. DAB color solution was added for color development, and the nuclei were stained with hematoxylin. Tissue sections were observed with light microscopy and photographed.

### Biochemical analysis

The blood samples were centrifuged at 3000 rpm at 4 ℃ for 15 min to separate the serum. The automatic biochemical analyzer was used to detect alanine aminotransferase (ALT) and aspartate aminotransferase (AST).

### Reverse transcription quantitative PCR analysis

Total RNA was extracted from liver tissue and cells using HP Total RNA Kit (Cat. R6812-02, OMAGE, USA). Reverse transcription was performed using 1 μg total RNA via a first-strand cDNA synthesis kit (Cat.04897030001, Roche, Switzerland). StepOne real-time PCR system was used to detect gene expression levels in liver tissues and cells.

### Western blot

Liver tissue or cells were lysed with RIPA lysate (Cat.R0020, Solarbio, China) at 4 ℃ for 30 min, and the supernatant was collected by centrifugation at 12,000 rpm/min at 4 ℃ for 10 min. Protein concentration was determined by BCA protein quantitation kit (Cat. 23225, Thermo, Germany). SDS-PAGE electrophoresis was performed with 50 μg protein per well, and the protein was transferred to PVDF membrane (Cat. IPVH00010, Millipore, USA). The PVDF membrane was sealed with 5% skim milk powder at room temperature for 1 h. Membranes were incubated overnight at 4℃. The following primary antibodies from Affinity Biosciences (Cincinnati, USA): α-SMA (Cat.AF1032), Collagen I (Cat.AF7001), FN (Cat.AF5335), β-actin (Cat. AF7018), GAPDH (Cat. T0004), IL-1β (Cat.AF5103), IL-6 (Cat.DF6087), E-cadherin (Cat.AF0131), Bax (Cat.AF0120), Bcl-2 (Cat.AF6139); The following primary antibodies from Bioss Antibodies (Peking, China): Integrin Alpha V + β6 (Cat.bs-5791R); The following primary antibodies from Cell Signaling Technology (Danvers, USA): Smad2 (Cat.3122), p-Smad2 (Cat.18338). After washing 3 times by TBST, the anti-rabbit IgG (Cat. ZB-2301, Zhongshan-golden bridge, China) and anti-mouse IgG (Cat. ZB-2305, Zhongshan-golden bridge, China) secondary antibodies was incubated at room temperature for 1 h. ECL chemiluminescence reagent (Cat. BL520A, Biosharp, China) was used for color rendering, and BIO-RAD gel imaging system and Image J was used for counting and statistical analysis of each band.

### Statistical analysis

GraphPad Prism 8.0 software was used to analyze all the experimental data obtained in this study. The data are expressed as the mean ± standard deviation. One-way ANOVA analyzed the differences among the groups. When P < 0.05, the data were considered statistically significant.

## Results

### Expression and purification of the recombinant LAP and tLAP

**Fig. 1:**
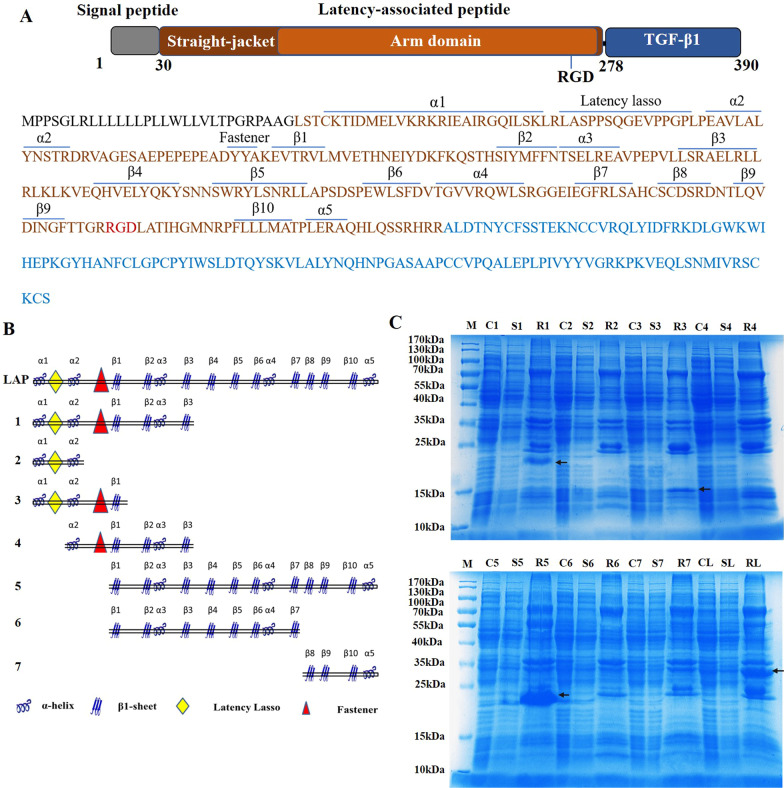
Truncated PET28a-LAP plasmid construction and expression detection. **A** Composition of the TGF-β1 proprotein (LAP: brown; TGF-β: blue). **B** Structural composition of truncated LAP plasmids. **C** Expression detection of truncated LAP. M, protein marker; C1–C7, Crushing bacterium fluid of No. 1–7; S1–S7, Supernatant of No. 1–7; R1–R7, Ni–NTA agarose of No. 1–7.CL, Crushing bacterium fluid of LAP, SL, Supernatant of LAP; RL, Ni–NTA agarose of LAP

Seven truncated recombinant plasmids were constructed (Fig. 1A, B). The expression results showed that: No. 1 and No. 3 had certain protein expressions, but the expressions were lower than that of full-length LAP. The expression level of No.5 was much higher than that of full-length LAP (Fig. 1C). The structural region in No.5 is relatively conserved among members of the TGF-β family. The α3 helix is associated with binding to TGF-β1, and the α5 helix, β1, β3 and β10 are involved in wrapping TGF-β1 to block receptor binding. Therefore No. 5 was named as tLAP and selected for purification and functional experiments.

**Fig. 2:**
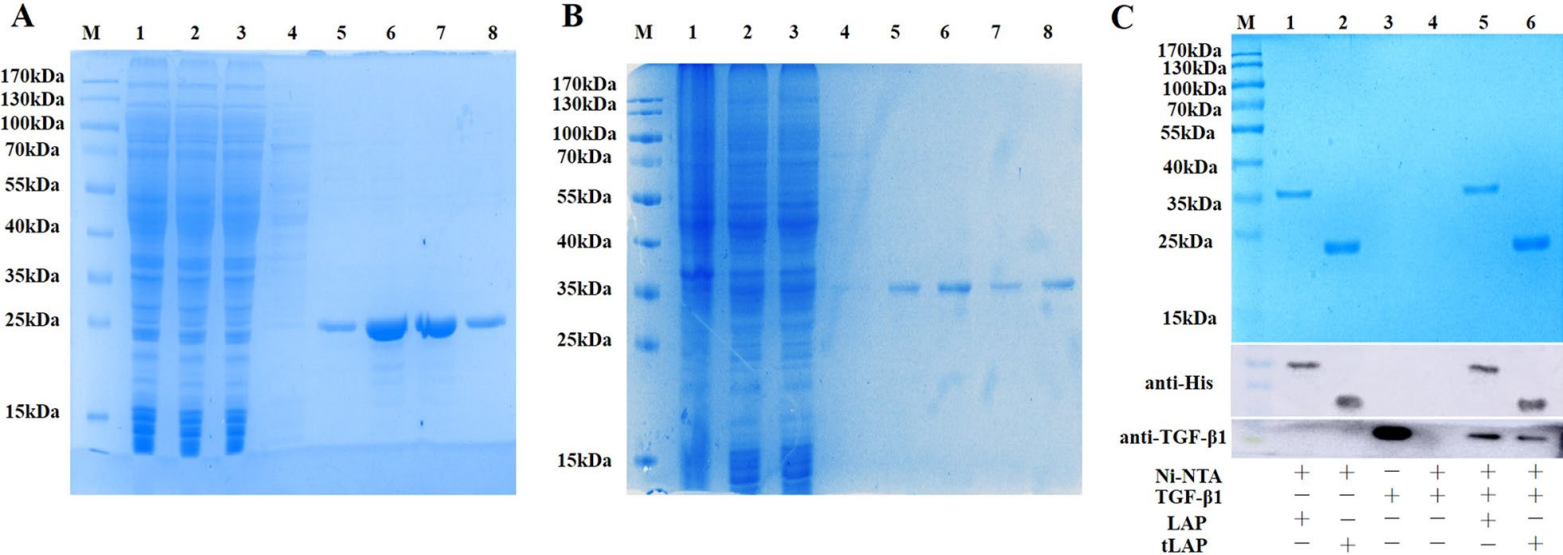
Purification and identification of Recombinant LAP and tLAP. **A** Purification of recombinant tLAP. lane M, protein marker; lane 1, Crushing bacterium fluid; lane 2, Supernatant; lane 3, Precipitation after crushing; lane 4, wash; lane 5–8, elution for 4 times. **B** Purification of recombinant LAP. lane M, protein marker; lane 1, Crushing bacterium fluid; lane 2, Supernatant; lane 3, Precipitation after crushing; lane 4, wash; lane 5–8, elution for 4 times. **C** Identification of recombinant tLAP by pull down assay. M, protein marker; lane 1, LAP + Ni–NTA agarose; lane 2, tLAP + Ni–NTA agarose; lane 3, TGF-β1; lane 4, TGF-β1 + Ni–NTA agarose; lane 5, TGF-β1 + LAP + Ni–NTA agarose. Lane 6, TGF-β1 + tLAP + Ni–NTA agarose

LAP and tLAP proteins were purified by Ni affinity chromatography. The results of SDS-PAGE showed that the expression level of tLAP was significantly higher than that of LAP. There are approximately 1.7 mg of LAP protein or 8 mg of tLAP protein in 1 L of bacterial solution (Fig. 2A, B). In previous studies, we demonstrated that recombinant LAP interacts with TGF-β1 in vitro (Song et al. 2022). We explored whether recombinant tLAP interacts with TGF-β1. The pull down results showed that TGF-β1 could not bind to Ni–NTA agarose alone (lane 4). When TGF-β1 was incubated with Ni–NTA agarose loaded with LAP and tLAP, TGF-β1 could be detected in Ni–NTA agarose (lane 5, lane 6), suggesting that recombinant proteins may interact with TGF-β1 (Fig. 2C).

### Recombinant LAP and tLAP inhibited TGF-β1-induced EMT, inflammation and apoptosis of AML12 cells

**Fig. 3:**
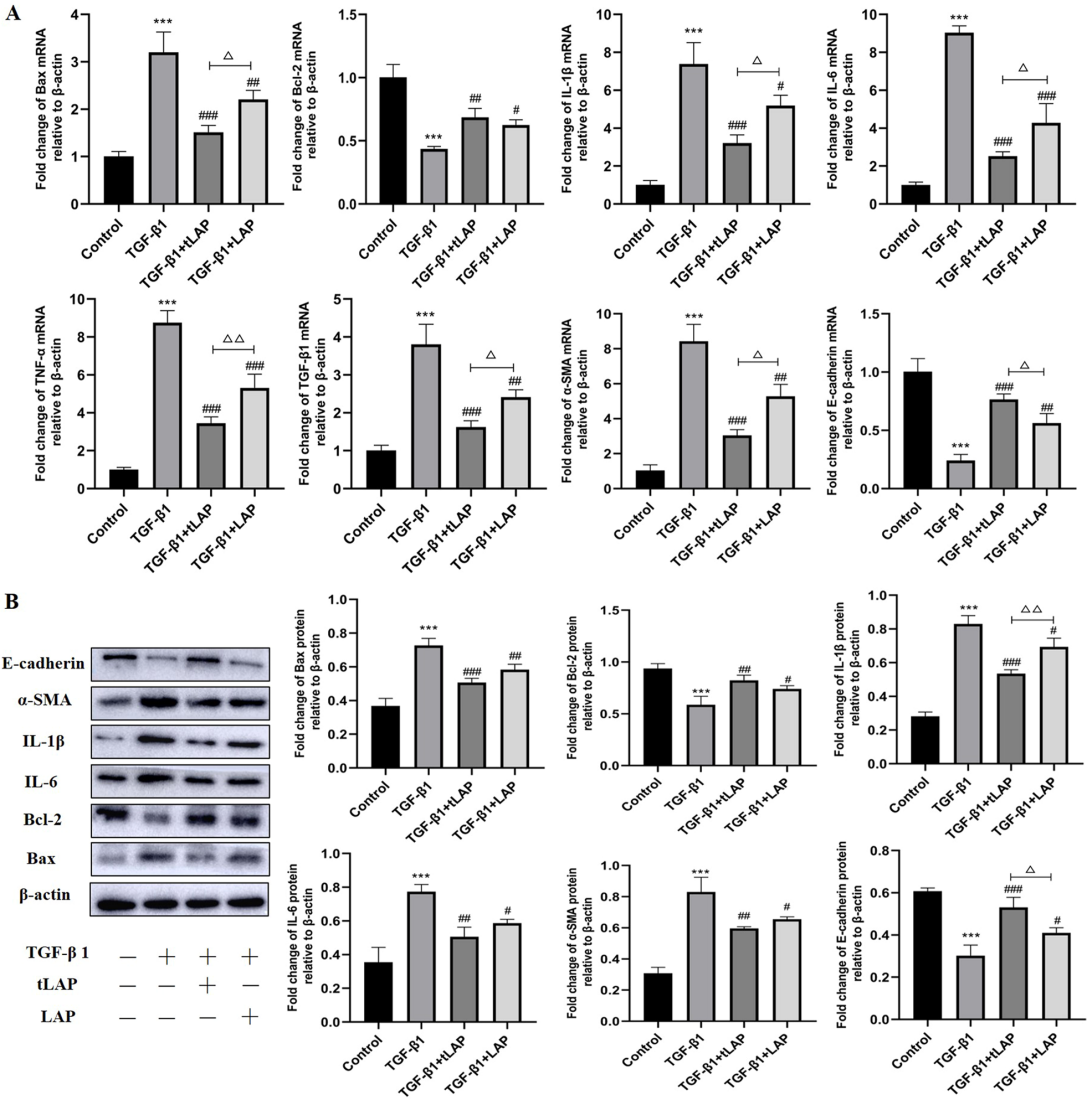
Detection of the effects of LAP and tLAP on TGF-β1-induced apoptosis and EMT in AML12 cells. **A** Detection of the mRNA expression of Bax, Bcl-2, IL-6, IL-1β, TNF-α, TGF-β, α-SMA and E-cadherin in AML12 cells. **B** Detection of the expression of Bax, Bcl-2, IL-6, IL-1β, α-SMA and E-cadherin in AML12 cells. ***P < 0.001 vs control group; ^#^P < 0.05 vs TGF-β1 group; ^##^P < 0.01 vs TGF-β1 group; ^###^P < 0.001 vs TGF-β1 group; ^△^P < 0.05, TGF-β1 + tLAP group vs TGF-β1 + LAP group; ^△△^P < 0.01, TGF-β1 + tLAP group vs TGF-β1 + LAP group; ^△△△^P < 0.001, TGF-β1 + tLAP group vs TGF-β1 + LAP group

To explore the effects of recombinant LAP and tLAP on TGF-β1-treated AML12 cells, we detected mRNA expression levels of apoptosis inflammation and EMT markers in TGF-β1 (5 ng/mL)-treated AML12 cells. RT-qPCR results showed that the mRNA expression level of E-cadherin was decreased in AML12 cells under the action of TGF-β1, while the expression level of α-SMA was significantly increased, these results suggest that AML12 cells lose the epithelial features and obtain mesenchymal phenotype by TGF-β1. In addition, the mRNA expression of IL-1β, IL-6, TNF-α and Bax in AML12 cells was significantly increased, while the expression of Bcl-2 was decreased. These results indicated that AML12 cells have inflammation and apoptosis under the action of TGF-β1. When treated with recombinant LAP and tLAP, these effects caused by TGF-β1 were inhibited (Fig. 3A). Western blot confirmed the expression of α-SMA, IL-1β, IL-6 and Bax in AML12 cells was significantly increased, but the changes in EMT, inflammation and apoptosis markers after TGF-β1 stimulation could inhibited by recombinant LAP and tLAP (Fig. 3B). Collectively, the results suggested that recombinant LAP and tLAP inhibited TGF-β1-induced EMT, inflammation and apoptosis of AML12 cells, and recombinant tLAP showed more effective inhibition than LAP.

### Recombinant LAP and tLAP inhibit TGF-β1-induced HSC-T6 cell proliferation

**Fig. 4:**
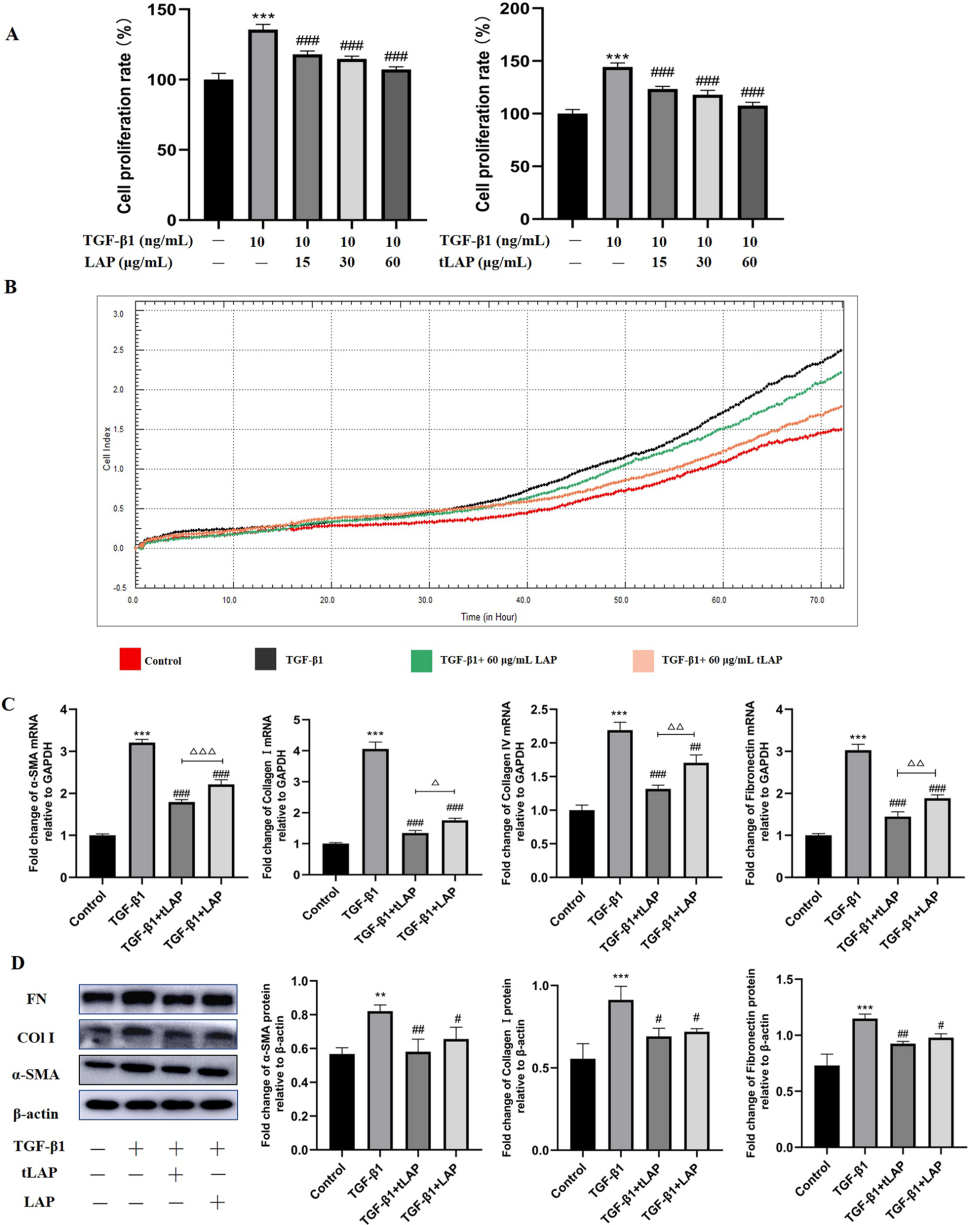
Detection of the effect of LAP and tLAP on liver fibrosis in HSC-T6 cells. **A** Detection of the effect of LAP and tLAP on cell proliferation by MTT. **B** Detection of the effect of LAP and tLAP on cell index by RTCA. **C** Detection of the mRNA expression α-SMA, Collagen I, Collagen IV, and Fibronectin in HSC-T6 cells. **D** Detection of the expression of α-SMA, Collagen I and Fibronectin in HSC-T6 cells. **P < 0.01 vs control group; ***P < 0.001 vs control group; ^#^P < 0.05 vs TGF-β1 group; ^##^P < 0.01 vs TGF-β1 group; ^###^P < 0.001 vs TGF-β1 group; ^△^P < 0.05, TGF-β1 + tLAP group vs TGF-β1 + LAP group; ^△△^P < 0.01, TGF-β1 + tLAP group vs TGF-β1 + LAP group

HSC-T6 cells were treated with 10 ng/mL TGF-β1 and different concentrations of LAP and tLAP. The MTT results showed that the proliferation of HSC-T6 cells significantly increased after TGF-β1 stimulation compared with the control group. In contrast, TGF-β1-induced HSC-T6 cell proliferation was significantly reduced after LAP and tLAP intervention (Fig. 4A). To confirm this result, the cell index (CI) of HSC-T6 cells was measured in real time every 1 min by using the RTCA system. The CI value of TGF-β1-induced cells was increased compared with the control group, the CI value of cells induced by TGF-β1 was decreased after LAP and tLAP treatment (Fig. 4B). Since the CI value is positively correlated with cell viability. Therefore, MTT and RTCA results indicated that LAP and tLAP could inhibited TGF-β1-induced HSC-T6 cell proliferation, and tLAP showed more effective inhibitory activity than LAP.

### Recombinant LAP and tLAP inhibit TGF-β1-induced fibrosis in HSC-T6 cells

**Fig. 5:**
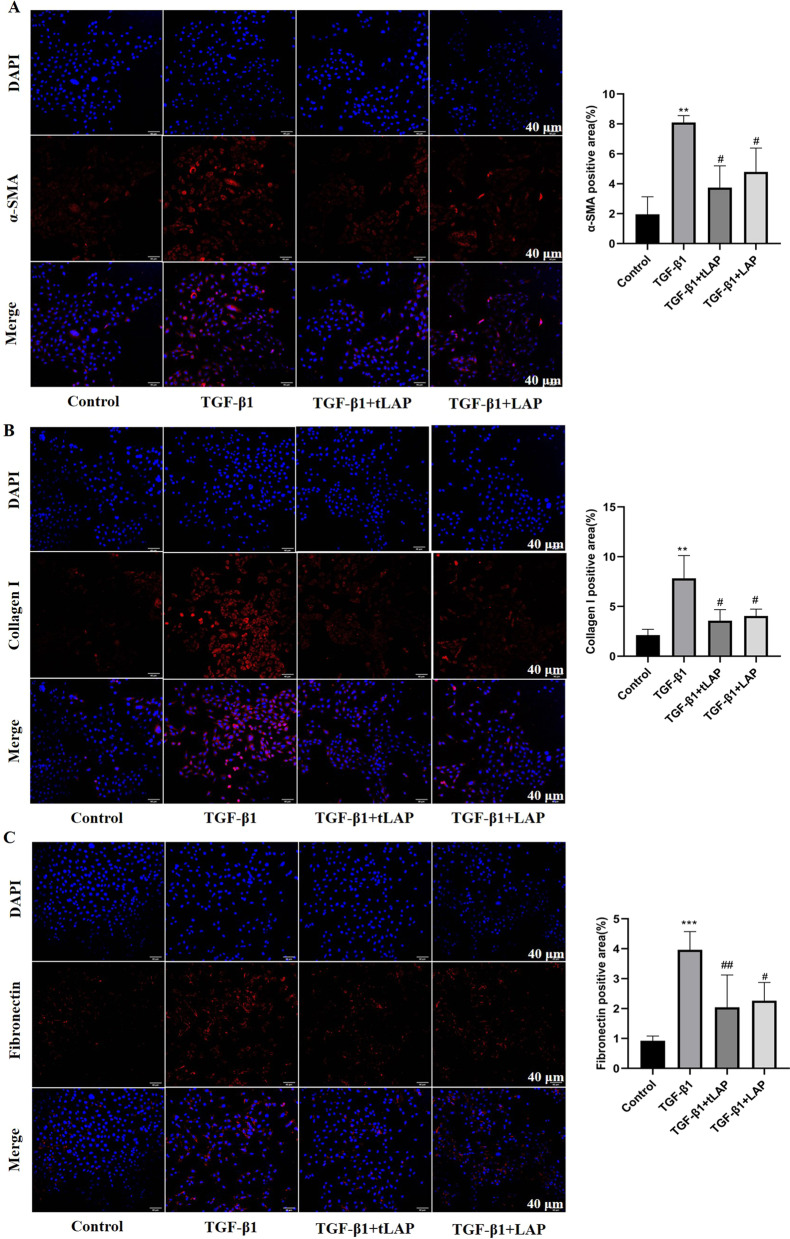
Detection of fibrosis-related proteins expression in HSC-T6 cells by Immunofluorescence. **A** Immunofluorescence detection of α‑SMA. **B** Immunofluorescence detection of collagen I. **C** Immunofluorescence detection of Fibronectin. DAPI (blue, nuclear stain) and antibodies to α‑SMA, Collagen I and Fibronectin (red), immunofluorescence staining (magnification × 200), Scale bars = 40 μm. **P < 0.01 vs control group; ***P < 0.001 vs control group; ^#^P < 0.05 vs TGF-β1 group; ^##^P < 0.01 vs TGF-β1 group

The effects of LAP and tLAP on the expression of fibrosis-related mRNA in TGF-β1-induced HSC-T6 cells were detected by RT-qPCR. The RT-qPCR results showed that the mRNA expression of α-SMA, Collagen I, Collagen IV, and Fibronectin in the TGF-β1 group were significantly increased compared with the control group, compared with the TGF-β1 group, the mRNA expression were significantly decreased by LAP and tLAP (Fig. 4C). The western blot results showed that compared with the control group, the expression of α-SMA, Collagen I and Fibronectin in HSC-T6 were significantly increased by TGF-β1 stimulation. Compared with the TGF-β1 group, the expression of fibrosis-related proteins were significantly reduced in LAP and tLAP groups (Fig. 4D). The immunofluorescence results showed that after TGF-β1 treatment, the fluorescence intensity of α-SMA, Collagen I and Fibronectin was significantly enhanced. The fibrosis-related protein fluorescence intensity decreased after LAP and tLAP treatment (Fig. 5A–C). The results showed recombinant LAP and tLAP can inhibit TGF-β1-induced fibrosis in HSC-T6 cells, and compared with recombinant LAP, tLAP has more effective anti-fibrotic activity.

### Recombinant LAP and tLAP alleviate CCl_4_-induced liver fibrosis in mice

**Fig. 6:**
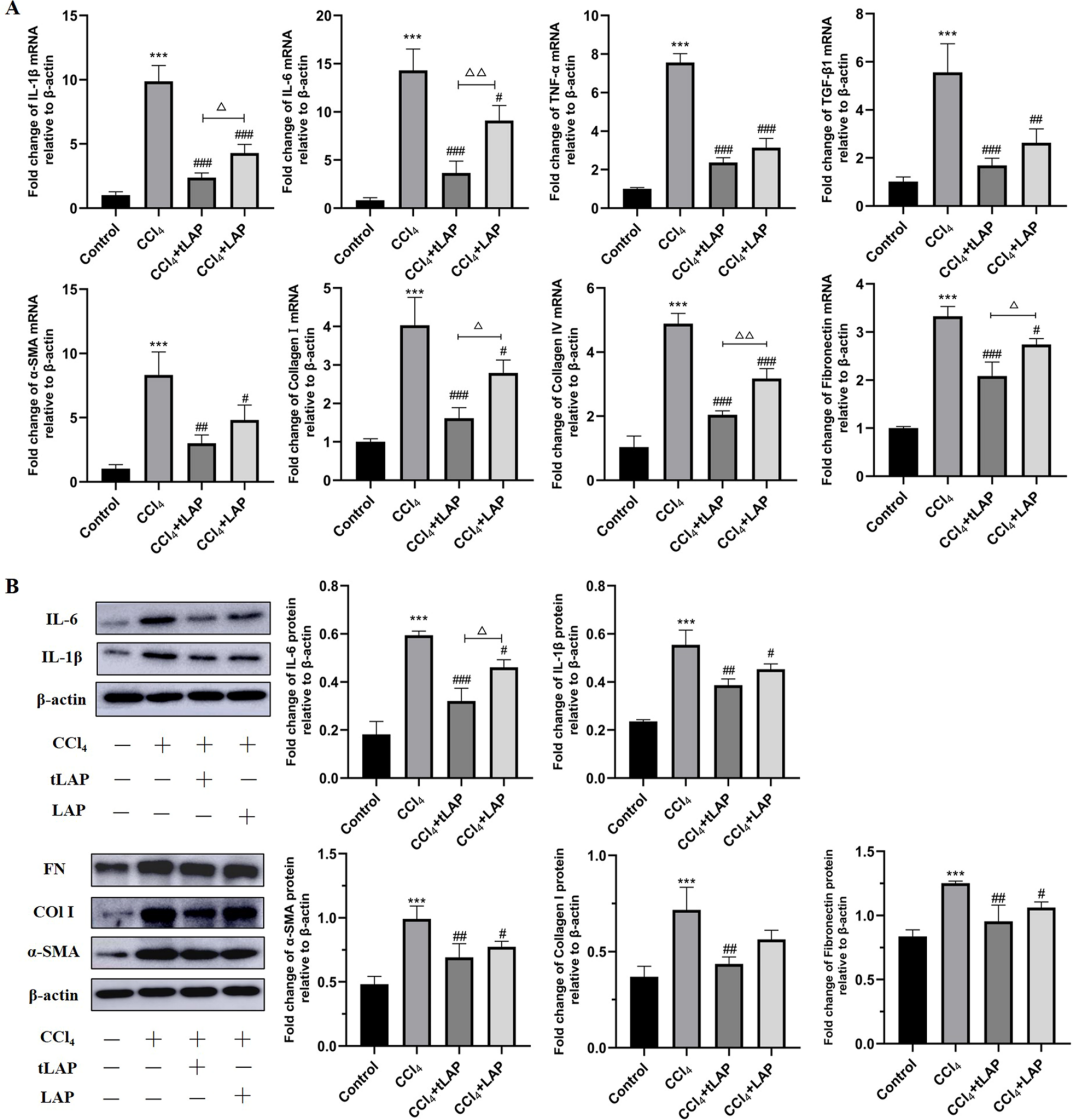
Detection of the effect of LAP and tLAP on fibrosis-related protein and mRNA expression in C57BL/6 mice. **A** Detection of the mRNA expression of IL-1β, IL-6, TNF-α, TGF-β, α-SMA, Collagen I, Collagen IV, and Fibronectin in C57BL/6 mice. **B** Detection of the expression of IL-1β, IL-6, α-SMA, collagen I and Fibronectin in C57BL/6 mice. ***P < 0.001 vs control group; ^#^P < 0.05 vs CCl_4_ group; ^##^P < 0.01 vs CCl_4_ group. ^###^P < 0.001 vs CCl_4_ group; ^△^P < 0.05, TGF-β1 + tLAP group vs TGF-β1 + LAP group; ^△△^P < 0.01, TGF-β1 + tLAP group vs TGF-β1 + LAP group

The anti-fibrotic activity of LAP and tLAP in vivo was observed by CCl_4_-induced liver fibrosis model in C57BL/6 mice. The mRNA expression of IL-1β, IL-6, TNF-α, TGF-β, α-SMA, Collagen I, Collagen IV, and Fibronectin in the CCl_4_ group were significantly increased compared with the control group, demonstrated liver inflammation and fibrosis. The recombinant LAP and tLAP could effectively inhibit the above gene changes induced by CCl_4_, and recombinant tLAP had more significant inhibition than LAP (Fig. 6A). Western blot confirmed that recombinant LAP and tLAP inhibited liver inflammation and fibrosis associated marker proteins elevation induced by CCl_4_ (Fig. 6B).

**Fig. 7:**
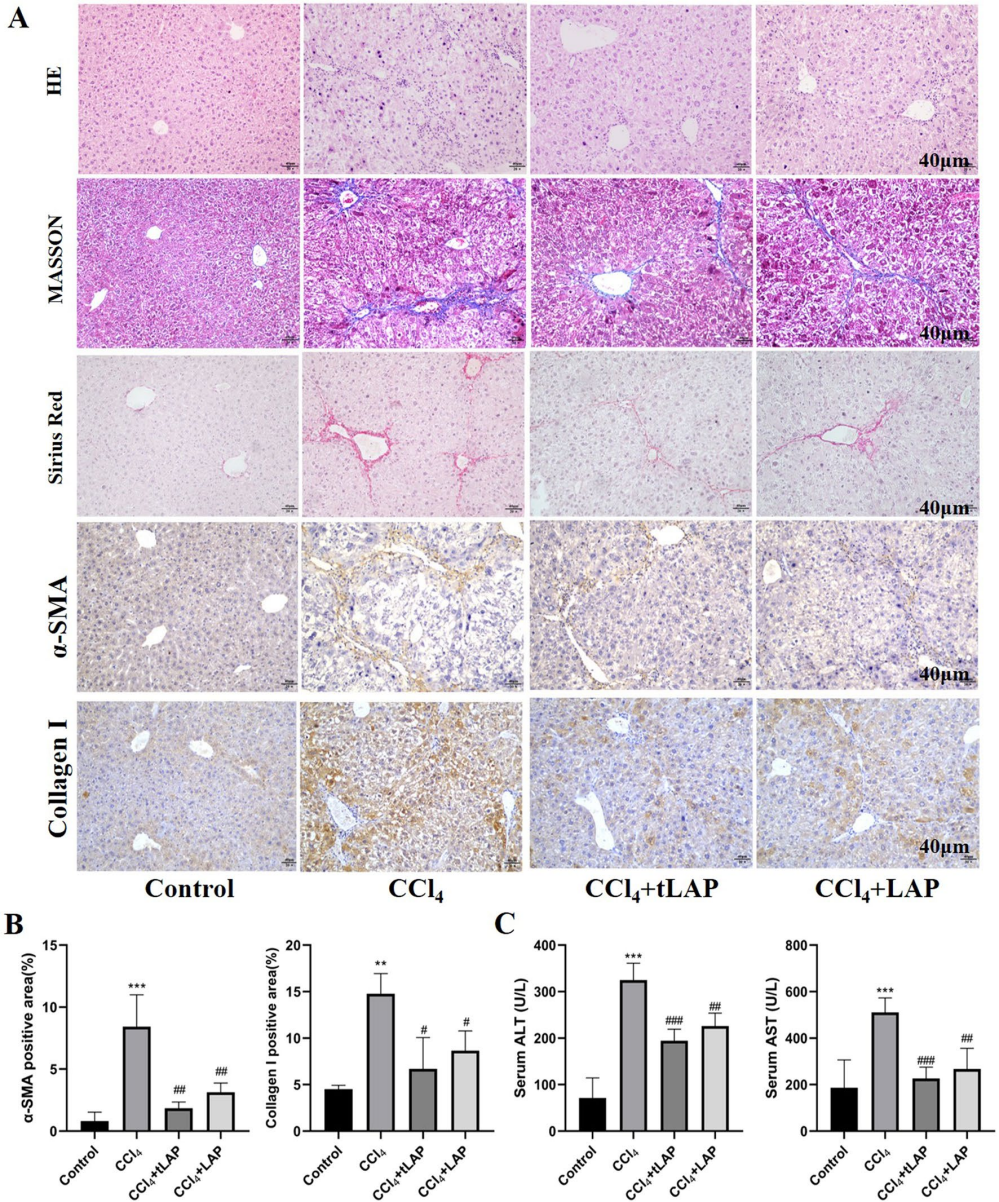
H&E, Sirius Red, Masson's Trichrome Staining and Immunohistochemical and Liver Function Indicators. **A** H&E, Sirius Red, Masson staining, and immunohistochemistry of the liver tissues. **B** Detection of serum ALT and AST activity in C57BL/6 mice. ***P < 0.001 vs control group; ^#^P < 0.05 vs CCl_4_ group; ^##^P < 0.01 vs CCl_4_ group. ^###^P < 0.001 vs CCl_4_ group

The results of H&E staining showed that the hepatocytes in the CCl_4_ model group had obvious inflammatory infiltration, deformation, and necrosis, which were alleviated in LAP and tLAP groups. Masson staining and Sirius red staining showed that compared with the control group, the liver of the CCl_4_ model group had a large number of collagen fiber hyperplasia, while the LAP and tLAP groups significantly decreased the fibrous hyperplasia. The expression of α-SMA and Collagen I in liver was analyzed by immunohistochemical staining. The results showed that compared with the model group, the expression of α-SMA and Collagen I were significantly decreased in the LAP and tLAP groups (Fig. 7A, B). Biochemical analysis showed that the serum ALT and AST of mice in the CCl_4_ group were significantly increased than those in the control group. Compared with the model group, serum ALT and AST were significantly decreased by LAP and tLAP (Fig. 7C). These results suggested that recombinant LAP and tLAP can effectively alleviate CCl_4_-induced liver fibrosis in C57BL/6 mice.

### Recombinant LAP and tLAP alleviate liver fibrosis via inhibition of TGF-β/Smad pathway

**Fig. 8:**
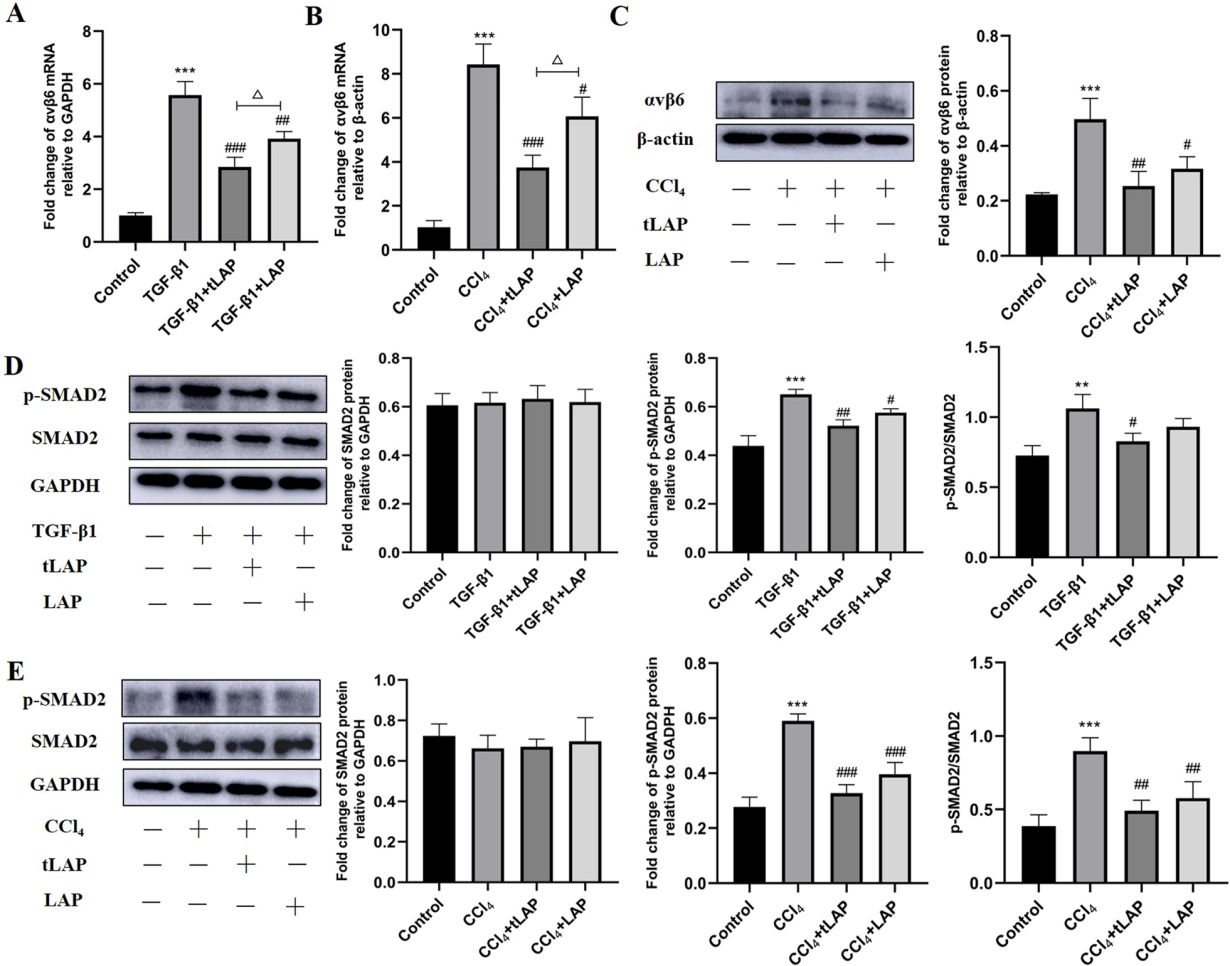
Recombinant LAP and tLAP alleviate liver fibrosis through TGF-β/SMAD pathway. **A** Detection of the mRNA expression of αvβ6 in HSC-T6 cells. ***P < 0.001 vs control group; ^##^P < 0.01 vs TGF-β1 group. ^###^P < 0.001 vs TGF-β1 group; ^△△^P < 0.01, TGF-β1 + tLAP group vs TGF-β1 + LAP group. **B** Detection of the mRNA expression of αvβ6 in C57BL/6 mice. **P < 0.01 vs control group; ***P < 0.001 vs control group; ^#^P < 0.05 vs CCl_4_ group; ^###^P < 0.001 vs CCl_4_ group; ^△△^P < 0.01, CCl_4_ + tLAP group vs CCl_4_ + LAP group. **C** Detection of the expression of αvβ6 in C57BL/6 mice. ***P < 0.001 vs control group; ^##^P < 0.01vs CCl_4_ group; ^###^P < 0.001 vs CCl_4_ group. **D** Detection of the expression of SMAD2 and p-SMAD2 in HSC-T6 cells. ***P < 0.001 vs control group; ^#^P < 0.05 vs TGF-β1 group; ^##^P < 0.01 vs TGF-β1 group. **E** Detection of the expression of SMAD2 and p-SMAD2 C57BL/6 mice. ***P < 0.001 vs control group; ^##^P < 0.01 vs CCl_4_ group. ^###^P < 0.001 vs CCl_4_ group

These results above indicated that LAP and tLAP could alleviate liver fibrosis in vitro and in vivo. However, liver fibrosis is a complex process regulated by multiple signaling pathways. LAP interacts with a variety of proteins in vivo. How to determine whether its anti-fibrosis activity is related to TGF-β1? Previous studies have found that LAP contains an arginine-glycine-aspartic acid (RGD) motif, making LAP a ligand for integrin αvβ6. Integrins bind to LAP through the RGD motif to regulate the activation of TGF-β1 (Thomas et al. 2002). Therefore, we examined the expression of αvβ6 in HSC-T6 cells and liver, and the results showed that the mRNA expression of αvβ6 was significantly increased in fibrotic HSC-T6 cells and liver, while recombinant LAP and tLAP inhibited the expression of αvβ6 (Fig. 8A–C). In addition, the effects of LAP and tLAP on the TGF-β1 downstream Smad pathway were investigated in HSC-T6 cells and liver. The results showed that the expression level of p-Smad2 protein was significantly increased in fibrotic HSC-T6 cells and liver, and recombinant LAP and tLAP could effectively inhibit the phosphorylation of smad2 protein. (Fig. 8D, E). These results suggested that recombinant LAP and tLAP may alleviate liver fibrosis via inhibition of TGF-β/Smad pathway.

## Discussion

When the liver suffers sustained injury, it often induces a self-repair response, leading to fibrous connective tissue deposition and eventually liver fibrosis. If not treated in time, most patients will progress to liver cirrhosis, liver failure, or even liver cancer (Ramadori and Saile 2004; Tanwar et al. 2020). Active and effective intervention is expected to delay or prevent the progression of liver fibrosis, prolong the survival of patients, and improve the quality of life of patients (Campana and Iredale 2017).

TGF-β1 is a highly pleiotropic cytokine with a wide range of biological functions. TGF-β1 signal transduction is involved in almost all stages of liver disease progression, including inflammation, fibrosis, cirrhosis, and even liver cancer (Fabregat et al. 2016). TGF-β1 promotes the activation of hepatic stellate cells into myofibroblasts and stimulates ECM synthesis (Shrestha et al. 2016). Studies have shown that serum levels of TGF-β1 are significantly higher in patients with liver fibrosis than in healthy people (Cayon et al. 2006). TGF-β1 is often secreted in the form of a latent complex. Most TGF-β1 is synthesized by parenchymal cells and secreted to the extracellular space, and the whole process is affected by a variety of factors. The signal peptide is cleaved before secretion. Under the action of enzyme, a complex composed of an N-terminal LAP and C-terminal mature TGF-β is connected by a non-covalent bond. Then, the complex becomes extracellular by changing pH, ion strength, or protease hydrolysis. Stripping LAP or changing LAP conformation releases mature TGF-β. When TGF-β binds to LAP, it cannot bind to its receptor (Clark and Coker 1998). The extracellular matrix glycoprotein thrombus reactive protein-1 (TSP-1) is one of the main physiological activators of TGF-β (Murphy-Ullrich and Poczatek 2000). TSP-1 activates TGF-β1 by binding to LAP, thereby disrupting the domain binding of LAP-mature TGF-β1 (Ribeiro et al. 1999). Thus, LAP removal is an essential step in TGF-β1 activation. Affecting TGF-β1 activation and receptor binding by LAP may be a potential treatment for liver fibrosis.

Considering the importance of LAP in TGF-β1 activation, LAP may be a key target for inhibiting the TGF-β1 pathway. In this study, we examined the anti-fibrotic effects of recombinant LAP and tLAP in vitro and in vivo. The anti-fibrosis activity of recombinant LAP and tLAP was confirmed based on the following results: (1) Recombinant LAP and tLAP inhibited TGF-β1-induced EMT, inflammation and apoptosis of AML12 cells; (2) Recombinant LAP and tLAP inhibit TGF-β1-induced proliferation and fibrosis of HSC-T6 cells; (3) Recombinant LAP and tLAP have protective effect on CCl_4_-induced liver injury and liver fibrosis; (4) Recombinant LAP and tLAP inhibit the phosphorylation of Smad2 protein.

In our previous study, the LAP-pET28a expression vector was constructed, and the recombinant protein was successfully expressed and purified. But the soluble expression of LAP was low, which was not conducive to large-scale production and application. Therefore, in order to improve its yield, based on the structural composition and function of LAP, a truncated tLAP-pET28a plasmid was constructed. By screening the induction conditions, we successfully purified the recombinant tLAP. The results showed that the yield of recombinant tLAP is nearly 5 times that of LAP.

We investigated the effects of recombinant LAP and tLAP on TGF-β1-induced AML12 cells. Our results showed expression of α-SMA, IL-1β, IL-6, TNF-α and Bax in AML12 cells treated with TGF-β1 were significantly increased and expression of E-cadherin and Bcl-2 were decreased, confirmed the occurrence of EMT, inflammation and apoptosis. Recombinant LAP and tLAP significantly improved TGF-β1-induced EMT, inflammation and apoptosis in AML12 cells. Then, we detected the effects of recombinant LAP and tLAP on the proliferation of HSC-T6 cells induced by TGF-β1 using MTT and RTCA. The results showed that recombinant LAP and tLAP can significantly reduce TGF-β1-induced HSC-T6 cell proliferation. Previous studies have shown that TGF-β1 treatment can activate the TGF-β/Smad pathway in HSC-T6 cells, leading to overexpression of fibrosis related genes and proteins (Gong et al. 2020). In this study, HSC-T6 cells were stimulated with TGF-β1 and treated with recombinant LAP and tLAP. The RT-qPCR,western blot and immunofluorescence results showed that recombinant LAP and tLAP could reduce the expression of fibrosis related genes and proteins in HSC-T6 cells induced by TGF-β1, and the results of RT-qPCR showed that tLAP had more effective anti-fibrosis activity compared to LAP.

In order to study the anti-fibrosis activity of recombinant LAP and tLAP in vivo. The liver fibrosis model was established by CCl_4_. It has been confirmed that CCl_4_ can induce liver inflammation, destroy liver cell membrane, cause liver lobule cell necrosis, and lead to liver fibrosis in mice (Unsal et al. 2021). AST and ALT are common markers of liver injury. Experimental results showed that recombinant LAP and tLAP could reduce the expression of ALT and AST in hepatic fibrosis mice. In addition, HE staining showed that recombinant LAP and tLAP could significantly reduce the infiltration and aggregation of liver inflammatory cells caused by CCl_4_, and MASSON and Sirius red staining showed that recombinant LAP and tLAP could significantly reduce the area of collagen fibers around the portal area of fibrosis liver. RT-qPCR and western blot analysis showed that recombinant LAP and tLAP could significantly inhibit the increased expression of fibrosis related genes and proteins in liver induced by CCl_4_, and consistent with the results of in vitro experiments, the recombinant tLAP protein showed more effective anti-fibrosis activity on the gene expression level.

Previous studies have shown that αvβ6 is related to the activation of TGF-β1, and LAP interacts with αvβ6 (Thomas et al. 2002). Therefore, the expression of αvβ6 was detected in this study. The results showed that recombinant LAP and tLAP can inhibit the expression of αvβ6 in fibrotic cells and liver. When TGF-β1 binds to HSCs membrane receptors and activates TGFβRI, the activated TGFβRI can induce Smad2 and Smad3 phosphorylation and combine with Smad4 to form smad2/3/4 complex, then transported into the nucleus together and regulates gene transcription (Dewidar et al. 2019). To determine whether the effects of LAP and tLAP on liver fibrosis are related to TGF-β/Smad pathways. We investigated the expression levels of Smad2 and p-Smad2 in HSC-T6 cells and liver. Results showed that p-Smad2 protein expression levels were significantly increased in fibrotic livers and cells, and recombinant LAP and tLAP inhibited Smad2 protein phosphorylation. These results suggested that recombinant LAP and tLAP may alleviate liver fibrosis through the TGF-β/Smad signaling pathway.

## Conclusions

In this study, we successfully constructed and prepared recombinant tLAP. The results indicated that recombinant LAP and tLAP could alleviate liver fibrosis via inhibition of TGF-β/Smad pathway. TLAP has higher expression level and more effective anti-fibrosis activity compared to LAP. This study may provide new ideas for the treatment of liver fibrosis.

## Data Availability

The data used and analysed during the current study are available from the corresponding author on reasonable request.

## Abbreviations

TGF-β1: Transforming growth factor-β1
LAP: Latency-associated peptide
HSCs: Hepatic stellate cells
RTCA: Real time cellular analysis
RT-qPCR: Real-time quantitative PCR
ECM: Extracellular matrix
SLC: Small latent complex
LTBP: Latent TGF-β-binding protein
LLC: Large latent complex
tLAP: Truncated LAP
ALT: Alanine aminotransferase
AST: Aspartate aminotransferase
